# The access paradox: abortion law, policy and practice in Ethiopia, Tanzania and Zambia

**DOI:** 10.1186/s12939-019-1024-0

**Published:** 2019-09-27

**Authors:** Astrid Blystad, Haldis Haukanes, Getnet Tadele, Marte E. S. Haaland, Richard Sambaiga, Joseph Mumba Zulu, Karen Marie Moland

**Affiliations:** 1 Global Health Anthropology Group, Centre for International Health, Department of Global Public Health and Primary Care, University of Bergen, Bergen, Norway; 2 Centre for Intervention Science in Maternal and Child Health (CISMAC), University of Bergen, Bergen, Norway; 3 Department of Health Promotion and Development, University of Bergen, Bergen, Norway; 4 Department of Sociology, Addis Ababa University, Addis Ababa, Ethiopia; 5 Department of Sociology and Anthropology, University of Dar es Salaam, Dar es Salaam, Tanzania; 6 School of Public Health, University of Zambia, Lusaka, Zambia

## Abstract

**Introduction:**

Unsafe abortion is a major contributor to the continued high global maternal mortality and morbidity rates. Legal abortion frameworks and access to sexuality education and contraception have been pointed out as vital to reduce unsafe abortion rates. This paper explores the relationship between abortion law, policy and women’s access to safe abortion services within the different legal and political contexts of Ethiopia, Tanzania and Zambia. The research is inspired by recent calls for contextualized policy research.

**Methods:**

The research was based in Addis Ababa (Ethiopa), Dar es Salaam (Tanzania) and Lusaka (Zambia) and had a qualitative exploratory research design. The project involved studying the three countries’ abortion laws and policies. It moreover targeted formal organizations as implementers of policy as well as stakeholders in support of, or in opposition to the existing abortion laws. Semi-structured interviews were carried out with study participants (79) differently situated vis-à-vis abortion, exploring their views on abortion-related legal- and policy frames and their perceived implications for access.

**Results:**

The abortion laws have been classified as ‘liberal’ in Zambia, ‘semi-liberal’ in Ethiopia and ‘restrictive’ in Tanzania, but what we encountered in the three study contexts was a seeming paradoxical relationship between national abortion laws, abortion policy and women’s actual access to safe abortion services. The study findings moreover reveal that the texts that make up the three national abortion laws are highly ambiguous. The on-paper liberal Zambian and semi-liberal Ethiopian laws in no way ensure access, while the strict Tanzanian law is hardly sufficient to prevent young women from seeking and obtaining abortion. In line with Walt and Gilson’s call to move beyond a narrow focus on the content of policy, our study demonstrates that the connection between law, health policy and access to health services is complex and critically dependent on the socio-economic and political context of implementation.

**Conclusions:**

Legal frameworks are vital instruments for securing the right to health, but broad contextualized studies rather than classifications of law along a liberal-restrictive continuum are demanded in order to enhance existing knowledge on access to safe abortion services in a given context.

## Introduction

This paper explores the complex relationship between abortion laws, policy and women’s access to abortion services with a focus on three different legal and political contexts. It is broadly acknowledged that legal frameworks are vital instruments for securing the right to health [1–3] but, as we will discuss in this paper, the connection between law, health policy and access to health services is complicated and critically dependent on the socio-economic and political context of implementation. Unsafe abortion, which is closely linked to restrictive abortion laws, and lack of access to contraception and safe abortion services, is a global problem of huge dimensions [3]. With an estimated 22 million cases annually, unsafe abortion is a major contributor to maternal mortality and morbidity [4], and between 4.7–13.2% of maternal deaths globally are estimated to result from unsafe abortion [5].

In sub-Saharan Africa, deaths due to usafe abortions have increased steadily since the 1990s [6], and the proportion of unsafe abortion to maternal death is estimated as high as 30% [7]. Young women in low income countries, and particularly adolescents living in rural areas, are disproportionately represented in the statistics [4] making unsafe abortion a massive inequity problem. Despite the scale and seriousness of the challenge, the controversial nature of the issue has pushed abortion to the margins of the global health agenda, and has made it one of the most neglected sexual and reproductive health problems in the world today [8]. Surrounded by stigma and neglect, the high rate of unsafe abortions has been referred to as a ‘silent pandemic’ [9]. The lack of access to safe and legal abortion is a major cause of unsafe abortion in many countries [8], but moral and religious contestations hinder political commitment and the legal reforms necessary to address the problem.

There is strong evidence that restrictive abortions laws do not reduce the prevalence of abortion [8]. Nonetheless, in most sub-Saharan countries, abortion continues to be illegal except to save the life of the pregnant woman, and criminal punishments are often prescribed for violations of these restrictions [3]. Conversely, a liberal abortion law is not sufficient to secure access to safe abortion services. Political will and the resources to finance and build services and to secure awareness about the services remain key elements [8]. Whether the law is liberal or restrictive, abortion is commonly stigmatized and frequently censured by political - and religious leaders, and a public stigma of abortion pervades local discourse in sub Saharan Africa. While both Christianity and Islam represent induced abortion as an act against the will of God, a broader global anti-abortion movement also condemns abortion on broader moral and philosophical grounds, arguing for the right to life of the unborn child. The moral stand against abortion has been challenged by the pro safe-abortion position, commonly defining safe abortion as part of Sexual and Reproductive Health and Rights (SRHR). The International Conference on Population and Development (ICPD) Programme of Action in 1994 [10] urged all governments and organizations to “strengthen their commitment to women’s health” and “deal with the health impact of unsafe abortion as a major public health concern” ([11], par 8.25). The following year, the Fourth World Conference on Women convened in Beijing moved one step further, stating that unsafe abortions threaten the lives of a large number of women, representing a grave public health problem. A plea to decriminalize abortion was agreed upon which referred to the ICPD Programme of Action for solutions [12]. In 1999 the ICPD+ 5 Conference Programme of Action moreover strengthened a call for post abortion care to recognize and deal with the health impact of unsafe abortion as a major public-health concern. The programme of action emphasized the importance of reducing the number of unwanted pregnancies through the provision of family planning counselling, information and services, and by ensuring that health services are able to manage the complications of unsafe abortion [13]. Post abortion care (PAC) - a concept initially launched by IPAS in 1991- was by and large not a very controversial concept and was gradually implemented globally. The diverse anti-abortion and pro safe-abortion movements and their attendant discourses, encountered on global, national and local levels, provide vital contexts for exploring the dynamics between national law and policy, and access to safe abortion services.

The study of the dynamics between national law and policy, and access to safe abortion services, requires critical engagement with discourses and movements for or against such access. Walt, Gilson and colleagues [14, 15] have for decades been preoccupied with the contextualization of health policy analysis, and have, not least, focused on how to carry out policy analysis in low and middle income contexts. In their writings they have addressed how the development and implementation of policy depends on the interplay between policy actors, context, process and content. They argue that there has been a tendency of researchers to focus on policy *content* alone, diverting attention away from the webs of *actors* who are located at the core of policymaking, the *processes* which explain why particular outcomes emerge or fail to emerge, and the particularities of the *contexts* within which policy is formulated and implemented [14]. Their ‘policy triangle framework’ is grounded in a political economic perspective, and considers how all of these elements (policy context and content, policy actors, and processes) interact to shape policy-making (ibid).

Similarly, calling for context-based public policy research focusing on the webs of actors who develop and implement policy, Wedel et at [16], Shore and Wright [17] and Shore, Wright and Però [18] criticise approaches in policy research that are based on the assumption of ‘policy’ as an orderly and legal–rational way of ‘getting things done’. They argue that such research is likely to miss out on the struggles over meaning, and the complexity of negotiation that are inherent to policy-making. The authors call for research that focuses on competing narratives and on socially produced and messy policy-making [18]. A central factor in making sense of the disorderly dimension of policy processes is that policy is no longer formulated primarily - or at least not only - by governments, but by a plethora of actors including supranational entities, NGOs etc. It may consist of webs of loosely connected actors located at multiple sites with varying degrees of institutional leverage [16, 18]. The differently positioned actors will be vested with different interests and power vis-à-vis the issue in question [19].

Inspired by such critical social science approaches to policy and policy implementation, which pay attention to the interplay between actors, contextual complexity and the ‘messiness’ of policy processes, this paper seeks to make sense of the articulation between abortion laws and policy, the implementation of such law and policy, and girls’ and women’s actual access to safe abortion services in three different contexts. In a comparative case study in Ethiopia, Tanzania and Zambia, we studied abortion-related law and policy documents, and explored how differently positioned actors within the field of abortion, interpret and act upon the (changing) landscapes of national abortion law and policy. The research is based on sub-studies within the Research Council of Norway funded project: *Competing discourses impacting girls’ and women’s rights: Fertility control and safe abortion in Ethiopia, Tanzania and Zambia* (2016–2018)*.*

### The three country contexts

Ethiopia, Tanzania and Zambia were chosen as cases for this study. They share a dominant anti-abortion discourse embedded in cultural and religious anti-abortion sentiments, but their legal frameworks for abortion are starkly different, making them interesting comparative cases.

The centrality of religion to people’s lives is a key feature of all three countries, and so is the role of religion in policy processes, not least pertaining to issues of sexuality and reproduction. In Zambia, where the churches have been influential since independence, the entangled relationship between politics and religion became particularly explicit when Zambia was declared a Christian nation in 1991 [20, 21]. In Ethiopia, the Ethiopian Orthodox Tewahedo Church is the largest (44%), with significant minorities of Muslims (34%), protestants and evangelical Christians [22], while in Tanzania approximately 35% belong to the Muslim- and 60% to the Christian communities [23]. Alongside powerful religious institutions with anti-abortion ideals and positions, an intricate web of national- and international institutions and organizations working to promote access to reproductive health services are present in all three countries. These organizations may be working for services in the entire country but are, by and large, based in the largest urban centres. These webs of actors commonly hold pro-safe abortion positions.

In spite of highly varied historical, social and political contexts, all three countries demonstrate high fertility rates varying from 4.1 in Ethiopia to 5.0 in Tanzania [24] and high maternal mortality ratios varying from 224 in Zambia to 398 in Tanzania [25–27]. The contraceptive use varies from 41% in Zambia to 25% in Tanzania, and among teenage girls it varies from 28% in Zambia to 9% in Ethiopia (cf table below).

The three countries share the problem of high unsafe abortion rates reflected in an extensive use of PAC services, but the social stigma associated with abortion and the difficulties of distinguishing between induced abortion and miscarriage cause data to be scarce and highly uncertain [28]. National data for Zambia are not available, but records from five major hospitals across the country between 2003 and 2008 show that almost one-third of all gynecological admissions were due to complications of unsafe abortion, and it is estimated that 6 in every 1,000 of these women died as a result of their complications [29]. In Ethiopia estimates indicate that despite the availability of legal abortion services, one third of adolescent abortions are clandestine and thus potentially unsafe [30]. There are strong indications that abortion related deaths have been reduced after the revision of the law in 2005, and recent estimates indicate that complications from unsafe abortions account for 19.6% of all maternal deaths [31]. In Tanzania where abortion is highly restrictive, no national data are available, and the incidence of unsafe abortion is hard to estimate since it is presumably hidden behind a high number of miscarriages. However, a study from 2013 [32] found that the majority of the abortions carried out were clandestine and a major contributor to maternal death and injury.

Globally, countries have been categorised by their abortion laws in diverse ways, commonly along a continuum from ‘severely restrictive’ to ‘liberal’ [33] or ‘prohibited altogether’ to ‘no restrictions as to reason’ [34]. At the restrictive end of the continuum are countries that prohibit abortion entirely or permit it only to save the life of the mother. Tanzania with its restrictive law permitting abortions only to save the life of the pregnant woman, sorts under this category. The law however includes explicit provision in the penal code exempting providers from punishment if they perform an abortion to save a woman’s life [35]. In the middle of the continuum lies Ethiopia which permits abortion to secure maternal life and health, and on grounds related to age and capacity to care for a child [36, 37]. Zambia is categorised in the liberal end of the continuum allowing abortion on both health-related and socio-economic grounds [38]. Countries classified in the very liberal end of the continuum having laws that permit abortion with few restriction, are not represented in our material (see [33]). The table (Table 1) below sums up the content of the three countries’ abortion laws, and key reproductive health indicators.

**Table 1:** Restrictiveness of abortion laws and reproductive health indicators

Country	Zambia	Ethiopia	Tanzania
Restrictiveness of the abortion law*	Abortion allowed to preserve physical or mental health and socio-economic reasons	Abortion allowed to secure maternal health and on grounds related to age and capacity to take care of a child	Abortion severely restricted and only legal to save the life of the mother
Maternal mortality ratio	224	353	398
Fertility rate**	4.9	4.1	5.0
Contraceptive prevalence girls 15–19***	28.1	8.9	14.9
Contraceptive prevalence 15–49***	40.8	28.3	34.4
Unmet need for family planning***	26.6	26.3	25.3

* Guttmacher Institute 2017. Abortion in Africa. Incidence and trends

https://www.guttmacher.org/fact-sheet/abortion-africa

**The World Bank 2017 https://data.worldbank.org/indicator/sp.dyn.tfrt.in

***WHO 2019 Global health observatory country views

http://apps.who.int/gho/data/node.country.country-ETH Last updated 2019-02-08

http://apps.who.int/gho/data/node.country.country-TZA?lang=en Last updated 2019-04-25

http://apps.who.int/gho/data/node.country.country-ZMB?lang=en last updated 2019-04-25

***Comparable and reliable data on abortion incidence not available

As we shall discuss in this paper the categorization of countries along a liberal-restrictive continuum based on their abortion law tells us very little about the reality of access to safe abortion services.

## Methods

Our research was based on qualitative exploratory research and had a cross-country comparative design. It involved studying 1) the historically and contextually embedded content of abortion law and policy, and 2) abortion-related policy processes through the exploration of ideas, positions and practical engagement in abortion-related work by actors within the field.

A review of central law and policy documents from the three countries was carried out in 2016–18. In addition, qualitative interviews were carried out with actors differently positioned within the field of abortion. This approach is well aligned with the classical call for ‘studying up’ in anthropology [39, 40], i.e. studying the views of powerful actors located in bureaucratic positions, in our case actors within ministries, NGOs and religious organizations. Six out of seven co-authors (AB, KMM, HH, GT, RS, MH) took part in at least one of four interview rounds with key stakeholders in Ethiopia, Tanzania and Zambia in 2016 and 2017. The article also draws upon abortion-related material generated in SAFEZT sub-studies by two of the co-authors (RS and MH).

Through discussions in the research team key policy institutions, organisations and actors within the field of abortion were identified within each of the three study contexts. Recognizing the important part played by actors beyond the government structure in policy making and implementation, we recruited actors from a broad spectrum of organizations and institutions. These included ministries, non-governmental organizations, UN agencies, professional associations and religious organizations representing diverse positions in the abortion debates. The list was expanded during the course of the research. By and large parallel organizations were recruited in the three countries (cf table 2 below). However, as it was deemed vital to represent different positions and prominent voices in the abortion debates in the individual countries, there is also a certain variation. We interviewed a total of of 79 individuals within the following categories: ministries (MIN), religious organisations (RO), non-governmental organisations (NGO), international non-governmental organisations (INGO), UN agencies (UN), professional organisations (PO), health workers (HW), journalists (J) and others (O). See table below (Table 2). In the manuscript we refer to the various actors using these abbreviation with Z for Zambia, E for Ethiopia and T for Tanzania.

**Table 2:** Overview of categories of actors included in the study

	Zambia	Ethiopia	Tanzania
Ministries (MIN)	4	3	4
Religious organisations (RO)	7	5	6
Non governmental organisations (NGO)	5	5	2
International non-governmental organisations (INGO)	4	8	5
UN agencies (UN)	0	3	1
Professional organisations (PO)	4	2	0
Health workers (HW)	0	0	6
Journalists (J)	2	0	0
Other (O)	2	1	0
TOTAL	28	27	24

A formal invitation letter was sent to the selected institutions/organisations. Nearly all individuals or organizations that were contacted agreed to take part in an interview, and some were re-interviewed after a year. The study participants were informed about the project in writing by email prior to the fieldwork and at the onset of the interview. Information was also provided about key research ethical principles. Written or oral informed consent was secured from all study participants. The study received ethical clearance from Regional Ethical Committee Western Norway, Norway (2017/1191) and data management clearance from Norwegian Centre for Research Data (57089/3/00SIRH); ethical clearance from the University of Zambia Biomedical Research Ethics Committee (009-07-17) and National Health Research Authority in Zambia (MH/101/23/10/1 and research clearance and registration from the University of Dar es Salaam (CoSS- SO18011). In Ethiopia the research was carried out following social science research procedures at Addis Ababa University.

The interviews were carried out in English and were guided by semi-structured and flexibly monitored interview guides. The informants were, by and large, articulate and actively engaged in the discussions. The topics raised included abortion policy, the role and activity of their organisation within the field of abortion, perceptions on their country’s abortion law and policy as well as on girls’ and women’s access to safe abortion services in their country. Most reflected critically on the topics raised.

The interview material was audio recorded, transcribed and analyzed throughout the data collection phase and during shorter intervals following each phase, with a comparative analysis taking place at the end of the data collection. During the comparative phase a full review of the material took place to gain an overview and to identify major patterns, including cross-cutting, contrasting or contradictory themes emerging in the material. The review was followed by the manual coding of the entire data set. Central content was subsequently moved into a separate document under headings which reflected the various dimensions of the main emerging themes. This document formed the basis for further analysis and write up of the material.

The team consisted of Ethiopian, Tanzanian, Zambian and Norwegian researchers, all employed at departments of social science or public health at national universities. All co-authors have substantial experience from long-term ethnographic research, primarily from research with a ‘reproductive health’ focus from eastern and southern Africa.

## Findings

We start each country section with the informants’ reflections on their national abortion laws, moving to their narration of the politics surrounding the abortion law and policy and their implementation, and finally to the perceived implications for access to abortion services.

### Zambia

#### The abortion law and the requirement for three signatures

As stated above, the abortion law in Zambia is classified at the liberal end of the continuum and The Termination of Pregnancy Act of 1972 [38] allows for abortions to be carried out on broad health and socio-economic grounds. However, according to our informants, there is an inherent ambiguity in the law that prevents it from granting women access to safe abortion services. The apparent contrast between what many referred to as a liberal law and severe difficulties of access was a key topic during our discussions with many stakeholders in Zambia, formulated and explained in the following manner; *yes in Zambia abortion is legal, the law is liberal, but for a client to access the abortion service is difficult* (INGO Z).

While the law does allow abortion on a broad set of grounds, it considerably restricts access to legal abortion services by requiring the signatures from three medical doctors, including a specialist. As a consequence, the law itself may be a barrier to access to legal and safe abortion services, especially outside the major urban centres where lack of qualified health personnel is particularly acute. One of our informants explained the barrier to access saying; *but when you go to a rural area, where are you going to get three doctors? Because in some rural areas you will not even have one doctor, so you find that it is difficult* (PPA Z).

The substantial challenge of securing the signatures was emphasized by informants we talked to from the NGO sector; *I am sure you know the picture in Zambia, we do not have sufficient health personnel, so already we a have limited number of doctors, but our law also offers conscientious objections. So the doctor…what if all three say, ‘I don’t want to participate in this process’, then you do not have anybody to consent for the services.* (INGO Z). When we enquired about the actual practice of securing the three signatures, informants emphasized that the demand was indeed maintained by the authorities, and that it implied a huge practical challenge for the providers. In addition to the conscientious objections, the difficulties include challenges related to transfers of doctors to different areas within Zambia and the movement of doctors out of Zambia; *yes they do practise it (the demand for 3 signatures), but you find that these same doctors withdraw…You find that the doctors are not there for a long period of time, they are moving* (INGO Z).

The temporary banning of the activity of an international NGO due to its apparent failure to secure the required signatures was brought up by our informants as evidence of the way the government follows-up this requirement; *In a number of cases when this process is not followed you have an uprising about not following the law. I am sure you have heard the story of what happened to Marie Stopes: it was banned because of the same three signature requirement* (INGO Z). *When they (the authorities) went into their institution to check, they discovered that they did not have the (signatures of) three doctors. That is how they were banned, until they were again legalized. Since that time you will not hear Marie Stopes talk about abortions in public* (INGO Z).

Beside the practical challenge of securing the signatures, it was pointed out that the procedure of obtaining the signatures was extremely costly for the INGOs involved in providing abortions. This requirement was considered to seriously restrict access to safe abortion services; *Access is very difficult, particularly for young, poor, rural girls* (INGO Z)*.* Informants within the ministries emphasized that the severe challenge of access to abortion services was valid beyond the rural areas, one explaining; *Actually, it is not just (difficult in) rural areas. I mean, the closest facilities to the women are the clinics and the health centres, and those hardly even have* *one* *doctor supporting the facility* (MIN Z)*.*

#### Zambia: a Christian nation

Representatives from different religious organisations in Zambia voiced their anti-abortion agenda in various ways. Some appealed to the protection of the unborn life as a gift from God; *We preach forgiveness and believe that a child is a gift from God so no life should be terminated. That is, a child can still grow into humanity and contribute to the nation… we believe that we are here to look after what God has given us* (RO Z). Other religious organisations would also speak out against abortion, but would argue that allowing abortions was an admission of failure in addressing the underlying economic and moral issues which caused unwanted pregnancies in the first place: *I think instead of talking about abortions, we should be talking about preventing abortions. What are the causes? Why do people want to abort a pregnancy that has been made?* (RO Z).

Looking back in time, the close connection between religion and politics became more evident when Zambia was declared a Christian nation. Indeed, the first time substantial questioning of the law took place was said to be during the early 1990s when President Chiluba came to power and presented the declaration; ‘*Zambia being a Christian nation, and (thus) the shame that goes with when you say ‘I have aborted’… It’s like you have no morals, you are a murderer* (NGO Z). We encountered anti-abortion sentiments and reference to the declaration beyond Christian organisations, for example among some employees in ministries; *As a Christian nation that is not a question to ask, because I won’t participate to help her terminate that pregnancy. We don’t do that* (MIN Z). The moral condemnation of abortion services was also said to have steadily increased among medical doctors; *now everybody was saying ‘why should we be terminating pregnancy when we are a Christian nation?’.. The doctors see themselves as Christians….* (INGO Z). Several of the informants explained that access to abortion services had been easier prior to the declaration; *I remember, when I was training as a nurse, we used to get a lot of clients coming for abortion services... I was working in the gyn department, it was very easy* (INGO Z).

A recent surge of attention arose in connection with the revision of the Zambian constitution; *I think one of the reasons why this has become an issue now was actually the constitution review process…. that sort of raised people’s eyebrows and people said ‘we have an opportunity to change this’* (INGO Z). A Bill of Rights stating ‘life begins at conception’ was, in this process, proposed as a constitutional amendment, a process in which many anti-abortion people participated. Although the Bill of Rights was not voted through because of a low turnout, the pro-safe abortion environment explained that they had been alarmed by the Bill and the referendum. Such an amendment could seriously undermine their work for access to safe abortion services. As proponents of safe abortion recounted; *We had a slow hiccup towards the election last year when that clause in the Bill of Rights almost went through.. it changed the focus to advocacy around the voting in the referendum*.. *The clause ‘life begins at conception’,- it was scary* (INGO Z).

Guidelines for safe abortion services in Zambia were developed in 2009, but they had not been efficiently disseminated and remained largely unfamiliar even to doctors. New and revised guidelines were in the process of being developed at the time of our fieldwork, but the future of the new guideline was deemed uncertain, again because of the anti-abortion movement dominating the political landscape. The limited knowledge about the existing guidelines among doctors was said to be paralleled by a lack of knowledge and misconceptions about the abortion law in the general population, related to the silence surrounding the topic. Our informants continually stated that the bulk of the population believed that the law was highly restrictive and that abortion was illegal in the country. The movement working to restrict access to abortions was said to be far larger than the movement working for increasing such access in Zambia. An INGO representative working in the field of abortion explained the situation like this; *They are backed by the Catholics, they are backed by other religious bodies and by the country’s President who relies on the Church for political mileage.. They have created a separate ministry for them [*i.e. *the Ministry of Religious affairs]* (INGO Z).

Despite the severe restrictions in access to safe abortion services - implied by the demand for three doctors’ signatures and the powerful anti-abortion discourse - informants readily addressed loopholes that, to some extent, would counteract the difficulties of access, at least in the capital Lusaka.

#### Referrals, Chinese clinics and misoprostol

It was explained that health personnel who did not wish to engage in abortion-related work, could refer patients to other health workers that would be able to provide help; S*ince Zambia is a Christian nation, you find a lot of nurses who want to affiliate themselves with religion. Then they say ‘you know what, I cannot do that’. But when that happens, the rule is that they refer this client to a nurse who can take her on. You do not deny (a patient) a service* (INGO Z). Informants also talked about the significance of increasing numbers of ‘Chinese clinics’ established in urban areas in Zambia that provided abortion; *You will find girls going to the Chinese doctors, because there you just go, and they tell you the amount of money and you pay. A lot of money, quite a lot of money* (NGO Z)… *What they do is that they start the abortion, and basically tell a woman to go to another facility to complete the evacuation. Or they do it themselves.. We rarely see prosecutions of them* (INGO). The more recent availability of Misoprostol and other combinations of abortive drugs in pharmacies was also said to be changing the landscape of access to abortion, at least in the urban areas; *The government procures misoprostol and they only procure it for haemorrhage, but everyone knows that girls have access to misoprostol over the counter in urban areas. In rural areas you find them in hospital stores* (NGO).

There were also indications that a certain level of lenience in terms of the demand for the three signatures was at times observed. The new guideline from 2017, signed by the Ministry of Health, was more liberal in its wording than the former. The challenge is, nonetheless, that the guideline has not been widely distributed and its contents are not known to the public. These factors illustrate the complex ways in which a permissive law is turned into policy and practice.

### Ethiopia

#### The radically revised abortion law of 2005

While still classified as illegal in the country’s Criminal Code, the revised abortion law of 2005 allows women to terminate pregnancies that result from rape or incest, if the fetus has a severe defect, or if a girl is under the age of 18 [41]. This implied a significant change from the previous act which allowed abortion only to save the mother’s life. An additional clause in the law states that the woman’s word is sufficient evidence of rape or incest, and the Technical and Procedural Guidelines for Safe Abortion Services affirms that ‘stated age’ is all that is needed to authorize an age-based abortion [37]. There was a consensus among our informants that there was high level of political commitment behind the revision of the law in 2005. The mandate given to the Ministry of Health to interpret and operationalise the law in procedural guidelines indicates this; *It (abortion) is not on demand, but if you look at the official interpretation of the law (referring to the Ministry of Health), more or less every woman who requests safe abortion care can access the service* … *As much as possible barriers to services are reduced. If she is a minor then no proof (is required) for age* (INGO E).

According to the ‘women-centred care’ concept underlying the guideline, eligible women have the right to access abortion services within three days of contact with the health services. Even more importantly, the additional clauses that the woman’s statement of rape or being underage is sufficient to obtain safe abortion services encouraged an understanding among many of our informants that abortion is almost permitted or legal; *At this time we don’t think about the limitation [in the law restricting safe abortion services]. It is actually allowed* (NGO E).

The law may be said to be restrictive as it is located within the country’s Penal Code, but although abortion is not permitted ‘on demand’, the law text backed by detailed clinical guidelines arguably make abortion rather liberal and permissive, particularly in a sub-Saharan African context. But, as we shall see below, roll-out and access to services are challenged by a number of factors related to the anti-abortion climate of the country.

#### Muted public debate in an anti-abortion environment

A problem pointed out by our informants was that the clauses that were intended to ease access to safe abortion services are not well known to the public or to individuals working in the police and judicial system. This was demonstrated during an interview with a senior officer in the Ministry of Justice, who argued that health workers would have to have a woman’s rape narrative proved by the justice system before an abortion could be carried out. According to informants in the INGO sector, police officers, set to investigate rape cases, were even less likely to know about these clauses in the abortion law. As a consequence, health workers performing abortion could be caught between the operational guidelines of the Ministry of Health, authorizing abortion solely based on a woman’s own claim of rape, and the demands of the police to have all rape cases reported.

Even with a far less restrictive law in place and a progressive guideline interpreting the law liberally, challenges to the implementation of the new policies were identified. Many related the challenges to prevaling religious-cultural norms; *We have seen that the law is not enough, putting in a guideline is not enough. Because this is a deeply cultured society with many religious and cultural interests* (PO E). Some of the liberal informants from the NGO-sector were concerned that in this highly conservative religious and cultural context, the government is careful not to step on anybody’s (religious) toes; *the government is reluctant to promote safe abortion.. the government wants the conservative people, religious people, to think abortion is illegal* (UN E).

Our interviews with priests and religious leaders from Islam, the Orthodox Christian Church and Evangelical Churches revealed, however, that they were very much aware of the law, and explicitly voiced their objections to it; *Until the synod changes its position on abortion, the revised abortion law will be interpreted as a transgression of our religious and cultural values related to the understanding of the honourable life of human beings* (RO E). Nevertheless, the religious organisations we interviewed had not taken an official stance against the revised abortion law.

The lack of discussion and publicity around the law made it possible even for the Patriarch of the Ethiopian Orthodox Church to ‘*look another way*’, avoiding open conflict with the government. One informant from the NGO sector recalled the Patriarch’s message to the policy-makers at the time of the revision of the law; *If you ask me officially, I would say no, therefore, just do it* (INGO E). However, engaging in public discussion to argue for safe abortion services was seen as risky including for those who were eager to protect the law; *if you come out to the media, it will bounce back* (UN E). *We don’t want to give them an entry point to revert it [the law]* (INGO E). Also one of the ministries emphasised that *We don’t share (information) with the media….we don’t talk about safe abortion* (MIN E). Engaging in promotion activity or activism, indeed, seemed to be avoided at all levels, and as our informants told us, it is vital to maintain the silence; *We don’t approach religious groups. Even when they have negative speeches, we don’t want to respond directly. We keep quiet* (INGO E).

#### Provider-negotiated access

The negotiated silence between religious, state and NGO-actors around the abortion law and safe abortion services had made it possible to avoid public confrontation and fuss around the law, but simultaneously limited the possibilities for dissemination, awareness creation and roll-out. As one of our informants in the NGO sector explained; *The services should be available at the health centre level, but only a few of them are providing the services. The law is here, but it is up to the NGOs to expand the services* (INGO E).

Despite the lack of access to abortion services at health centre level, the expansion of the service was, in general, said to be clearly substantial. This achievement was said to be crucially dependent on the large-scale training of mid-level providers in first trimester safe abortion care at primary level, and this training was said to take place on a large scale. The task-shifting from medical doctors to nurses and midwives was seen as vital in this context because of severe doctor shortages. At the same time, as some informants emphasised, health workers continue to be gate keepers to the services and a lot is left to their discretion; *I still feel that it is under the jurisdiction of the health professional to interpret and apply the family [abortion] law. There are some listed conditions to terminate a pregnancy, but there is also a gap that any professional who keeps the interest of the client can use. Definitely that depends on the attitude of the professional. If you want to use it, you will use it in the best interest of the client, but if you don’t that is also possible* (PO E). Health workers could refuse to provide abortion services for moral and religious reasons, or - in the context of transparent and anti-abortion dominated rural communities - to avoid being marked out as an ‘immoral’ abortion provider. The transparency of local communities also prevented women and especially young girls seeking abortion from public health facilities. Women were said commonly to prefer accessing safe abortion services in a private hospital while the public health centres were vacant. Cost was, however, perceived to be a big challenge in private health facilities.

Cost was also a central topic in relation to the recent scale-up of medical abortion. Medical abortion was said to simplify procedures and to improve access to services for women who can pay, but not for the poor since; *medical abortion drugs are not free in the country, so wherever you go the client has to pay for the drug. So when a young girl of 15.. who does not know even where to go to have a safe abortion is requested to pay for the drug - how can she afford it?* (INGO E). While some of our informants claimed that clients could only buy the drug in private clinics and NGO clinics, others claimed that Misoprostol was available in pharmacies and sold illegally over the counter. The latter suggested that the health authorities, though well informed about the illegal sale, chose not to act upon it. As an informant in one of the ministeries told us; *We are not punishing the sale [of Misoprostol] because it helps the woman. If the girl goes for traditional ways it will cost her. It is better to go to the pharmacy* (MIN E). This tacit tolerance for the illegal marketing and use of Misoprostol was thus justified by the risk of severe complications of other clandestine procedures. As pointed out by a medical doctor who had experienced the reduction in septic abortion and death during the last decade; *The tragedy* [of septic abortion] *has almost ended* (INGO E).

As we have seen in this section, access to safe abortion services in Ethiopia, though vastly improved after the revision of the law and the dissemination of the procedural guidelines, is still limited by the silence surrounding the law and the subsequent lack of advocacy and information about the services. This makes health workers powerful gatekeepers of safe abortion services.

### Tanzania

#### Within the frames of a highly restrictive law

The abortion law in Tanzania has remained highly restrictive with the penal code stating that termination of pregnancy is legally permitted if it is performed to save a woman’s life [35]. However, in contrast to the complicated procedures in Zambia requiring the signatures of three medical doctors including a specialist, the Tanzanian abortion law does not specify the level of provider who can decide on eligibility. In practice, a mid level health worker like a midwife can perform an abortion without consulting others [42]. Efforts to liberalise the law have met strong opposition and the last attempt in 2012 failed. Like in Lusaka and Addis Ababa, there are a number of NGOs and UN organisations working to extend safe abortion and post-abortion care (PAC) services to women and girls in an anti-abortion political and cultural context. Representatives from the NGOs we interviewed stated that the law should be changed because of the implications of the vast numbers of unsafe abortions; *I think the law should be revised, we have seen the negative side (of the law) for too long. They should revise both the sex education in schools and also look at legalizing safe abortion. Mmmh, − this is not to say that one would kind of promote abortions, but we will have to face reality; people have abortions, it is happening at a large scale..* (INGO T). Another informant similarly stated; *there is a need to change the law I think, because.. you know within the health services you often interact with clients who have induced an abortion and they have come to you for services, so I think the law has to be changed* (INGO T).

The actors interviewed did, however, emphasize the difficulties related to advocating against the restrictive abortion law. As one informant phrased it; *I think in Tanzania the push for a revision of this law is very little. It is a very sensitive issue* (UN T). A representative from an NGO providing PAC services, made it clear that her organisation could and would not be involved in advocacy to change the law; *We have always made sure that we stay away from the legal and the rights aspect. We communicate to the clients that we don’t advocate for your rights, but we can provide services to you with dignity and respect, regardless of what you do and how you choose to live. But we cannot be used as the platform to advocate for things that are against prevailing laws* (INGO T).

The severe challenge of finding entry points to make the abortion law more liberal within the present policy landscape in Tanzania was brought up by informants; *It is very difficult to find an angle, because if other stakeholders are coming in... there is a lot of resistance from the religious groups, because committing an abortion is considered killing, so it is very difficult to argue* (UN T).

#### The tension between the different positions on abortion

Attempts to introduce changes to the law have nonetheless taken place in the Tanzanian context, largely driven by the Tanzanian Women’s Lawyer’s association (TAWLA) and through recent moves towards the East African Community with reference to Tanzania’s human rights obligations. In 2012, a network of local and international NGOs namely, TAWLA, Care International and the White Ribbon Alliance, made moves towards a pro-safe abortion legislation. These actors jointly produced a bill of law which ended at the parliamentary caucas responsible for safe motherhood, but they did not succeed. The only concrete advocacy strategy considered feasible by the organisations we interviewed was ‘*to follow the health track*’; i.e. to argue for a change in the law by using public health arguments. However, the lack of knowledge about the actual health outcomes or burden of unsafe abortion, for example in terms of maternal mortality numbers, was brought up as a major barrier to such a stratety: *If you don’t have strong evidence or a basis for your arguments (it is difficult).. Professionals like gynaecologists can come up with data on the number of people who are dying and the complications, and they can present it in a way where it can make sense to the politicians* (UN T).

As in Zambia and Ethiopia, the need to be closely aligned with the government and to move very carefully when discussing the abortion issue was strongly emphasized by the organisations working for access to safe abortion services; *We have our entry point through the government. When you engage them from the beginning they will give you support. If you don’t do that, expect huge resistance from the leaders; councillors will just resist.* (INGO T). The immense public sensitivity about abortion and the way to move forward was stressed repeatedly by the organisations working for access to safe services; *In the introduction, if you mention that you will do safe abortion work they (community members) will chase you, but when you go (to the communities) and say that you will do prevention of unsafe abortion…* so *we will always give them what they want to hear* (INGO T).

On the anti-abortion side such attempts to step carefully were ridiculed. *A representative of a group that referred to itself as a ‘pro-life movement’ spoke at length about how his organisation had engaged in campaigns among others against TAWLA’s move to change the law*, and mentioned various examples; *The idea was for countries to ratify and domesticate the law [the Maputo Protocol], and integrate it into the existing policy as ‘Safe Motherhood’. Sounds sweet, but it is about abortion and contraception… When pushing for abortion in Tanzania, the advocates had to redefine it, using the language of reproductive health. But this could not be pushed through in the constitution, so they now try to push their agenda through the block of the East African Community. But this [pro-choice community] cannot enforce law at national level…. At times they talk well, but it is the words of the devil* (RO T).

When he was informed about our project’s work in Tanzania, Ethiopia and Zambia, the informant above commented on what he referred to as *the pro-life foundations at the grass roots* in the three countries; *The law in Ethiopia is free (liberal). There we have the will of the people against the government. The people are not ready for the liberalization. If you talk against abortion (in Ethiopia) you are talking against the government, but the heart of the people is still pro-life. We see the same in Zambia. People were not ready, and Marie Stopes were driven out of Zambia. The spirit is pro-life* (RO T)*.*

Despite the severely constraining legal and policy environment and the religious and culturally grounded attitutes prevalent at the grassroots, we thus also in a Tanzanian context encountered some avenues that allowed access to relatively safe abortion.

#### Illegal provision, limited prosecution and access to misoprostol

While the Tanzanian law and political discourses signal a highly conservative and restrictive abortion scenario, our informants simultaneously communicated what emerged as very real avenues of access to relatively safe abortion services, particularly in Dar es Salaam and other larger cities. Prosecutions of both girls or abortion providers could be encountered, but with the abortion rates in mind, prosecutions emerged as extremely rare. Indeed, informants from the NGO sector generally held that abortions were not prosecuted in the country; *There are no convictions, no one prosecutes and no one reports it. When people don’t report, the law becomes dormant* (INGO T).

A pragmatic approach to reporting of abortions within the health system was similarly communicated, one informant saying; *In the hospitals, if they come, even when you find out that this person has had an abortion, usually they won’t report it anywhere. You will just treat the patient and that person will go home*... (UN T). Another informant similarly stated that you need to help and not prosecute girls; *Because it’s almost unfair to unilaterally target clients.. They have broken the law yes, but at that point what you need is to provide services. Any aversive action can make them run away from the health facilities that they should be running towards saving their lives. So I don’t know if anyone or any girls have been prosecuted for attempting an abortion* (INGO T).

The fact that only one signature is demanded by the law to allow a health worker to carry out an abortion was said to leave considerable room for doctors to define when a mother’s life is in danger. According to the NGOs there was substantial willingness among health workers to assist a woman in need of an abortion; *In Dar es Salaam for instance there are over 250 health facilities that provide reproductive and child health services, and there are close to 500 health facilities that provide all sorts of health services. And you know all these health facilities... they get an umbrella to provide services that are not listed on their licence, including abortion services* (INGO T). Some abortion providers working outside the health system were said to refer women to a public health institution after carrying out the abortion; *When I was working in a clinical setting, what you would observe is that they would perform an abortion and tell the client to go to a public health facility (after the abortion), because they know ‘post-abortion care’ is available there. So you get a lot of post-abortion clients because the abortion was induced for you...* (INGO T).

Furthermore, a small revolution was said to have taken place in terms of the rapidly increasing availability of medical abortion in the country. But the secrecy surrounding the sale of Misoprostol was emphasized with reference to the legal and policy environment in the country; *If you go to the medical stores in the streets selling Miso… For which purpose people are buying them they won’t tell you. Because of the situation of the law people are not exactly open.., because when they find you are having an abortion it is an issue in Tanzania* (NGO T).

We thus encountered a highly conservative abortion-related legal and policy environment in Tanzania, but also avenues that made it possible for health care providers and abortion-seeking women to navigate access to relatively safe abortion services. With a general lack of reporting and prosecution of abortion cases, an openness among certain health workers to performing and hiding abortion cases combined with a surging ‘miso-market’, a scenario of certain possibilities of access to services emerged in urban areas despite the severely restrictive legal context in the country.

## Discussion

### Abortion laws and access to abortion services

This study has aimed to shed light on the complex web of factors that mitigate the relationship between law, policy and practice, questioning assumptions about the law in order to understand actual access to services.

In line with Walt and Gilson’s [14] call to move beyond a narrow focus on the *content* of policy, our study demonstrates that a broad classification of abortion laws along the liberal -restrictive continuum (see e.g. [33]) has limited value in terms of understanding women’s access to safe abortion services. This study’s findings reveal that the law texts that make up the three national abortion laws are highly ambiguous. The Zambian abortion law at the liberal end of the continuum, permitting safe abortion on broad social and medical grounds, is profoundly affected by the requirement for three consenting signatures by medical doctors, which is hardly possible to obtain even in hospitals in urban areas. By contrast, in Ethiopia, the abortion law is placed under the Penal Code signalling that abortion is illegal, while at the same time the law and policy guidelines state that a woman’s word that she was raped, a victim of incest or is underage is enough for her to qualify for legal abortion. In theory, these clauses make abortion widely accessible although, as we have seen above, the reality is more complex. Finally, in Tanzania, the highly restrictive status of the abortion law is mitigated by what seems to be a lenient attitude to both provider-induced and self-induced medication abortion and limited prosecution of illegal abortion-seekers and providers. Interestingly, the text of the law ‘to save the life of the mother’ and the demand for only one health worker to decide on whether the abortion-seeking woman is eligible for legal abortion services, leaves a lot of discretionary power with the individual health workers.

Hence, in our three country cases, the relationships between law texts and actual access to services appear to be rather paradoxical. In order to enhance the understanding of such apparent ambiguities and paradoxes, we will, in line with Walt and Gilson [14], turn our attention to the *contexts* within which central abortion-related *actors* and *processes* are operating.

### Locating abortion laws and actors in context

Throughout our study, we met *actors,* differently positioned, with different interests in abortion as a religious, public health or rights issue, and with different powers to make their position relevant to the public and to policy process.

Despite the conservative, anti-abortion environment in Ethiopia where the Ethiopian Orthodox Christian Church continues to enjoy cultural power and to a large extent shape public opinion in moral matters [43] the restrictive abortion law was replaced by a law that drastically expanded the grounds on which women could access legal abortion. The revision took place in an alliance between a number of *civil society actors* and *key actors* in the Federal Ministry of Health, promoting safe abortion as a public health measure to reduce maternal mortality. To understand how this radical move could happen, we need briefly to remind the reader of the Ethiopian political *context* which has been characterized by powerful and authoritarian regimes with substantial power to fight through their agenda. At the turn of the century, the regime put the Millenium Development Goal (MDG) on maternal health (MDG 5) high on the political agenda. Avoiding a tenuous framing of induced abortion as a women’s rights issue, the governmental discourse gained legitimacy through the aim of protecting girls and women from the adverse health implications of unsafe abortions, and ultimately of reducing abortion-related death rates [6]. In the contentious climate of the revision of the law, the abortion law was retained within the penal code, signalling to the public that abortion is illegal in Ethiopia, while the Ministry of Health was mandated to make safe abortion services accessible. Operating within this tension between the religious communities’ concern about the sanctity of life and public health aims, the government installed the most permissive abortion law in Eastern Africa. In the past decade, the Government of Ethiopia has demonstrated its capacity to implement the abortion policy and increase accces to safe abortion services on the basis of its public health ideology despite low popular support. The approach, as we have indicated in this paper, has been low profile and ‘silent’ in order not to create resistance. The scaling up of services and the implementation of safe abortion care has therefore probably been slower than a fully open approach would permit, but with an open approach the risk of backlash would have been higher.

In Zambia, the public stand has been intimately connected to the religious-moral dimensions of abortion, representing induced abortion as a moral and legal offence. As seen above, Zambia declared itself a Christian nation in 1991, and religious language has become the language of politics in the country. Indeed a conservative Christian discourse has, through Zambia’s recent history, become increasingly powerful and has emerged among others in a subtle anti-abortion discourse. The recently proposed Bill of Rights that included a clause stating that the right to life begins at conception, potentially with vast implications for abortion-seeking women, revealed the political strength of the anti-abortion actors. This process brought the abortion issue, that had been silenced, back onto the political agenda and constrained the political scope of action for actors working to liberalise the law.The constitution review process was seen by some civil society actors as a tactic to conceal the abortion issue among the series of progressive rights proposed in the bill. This process highlights the restrictive nature of the abortion environment in Zambia with consequences for women’s access to comprehensive reproductive care [44].

In Tanzania where Islam and Christianity are practised by the vast majority of the population, the presentation of abortion as a sin and as a moral transgression predominates public discourse. Although Tanzania ratified the African Charter’s Protocol on the Rights of Women in Africa [45] (also referred to as the Maputo Protocol), which requires the government to “protect the reproductive rights of women by authorising medical abortion in cases of sexual assault, rape, [and] incest, and where the continued pregnancy endangers the mental and physical health of the [pregnant woman] or the life of the [pregnant woman] or the foetus.”(p. 16), it never domesticated it. Nevertheless, in the context of high maternal mortality rates and with the aim of achieving MDG 5 on maternal health, the government and allied actors within the large international NGO sector have taken important steps to reduce maternal mortality including deaths caused by unsafe abortion. This is spelt out in the National Road Map Strategic Plan to Accelerate Reduction of Maternal, Newborn and Child Deaths in Tanzania, 2008–2015 [46]. Furthermore the Tanzanian Food and Drugs Authority (TFDA) approved the use of misoprostol for the treatment of incomplete abortion in 2011 [32] which spurred a demand for Misoprostol off label. The Government has not acted upon this development despite the fact that it has been debated in the media. Hence, the context of implementation of the abortion law is multifaceted and implementation of the law seems to be an expression of this. (The three country cases are elaborated in separate case studies in this thematic issue/series.)

### The messiness of abortion policy and the paradox of access

As demonstrated at the start of this paper, Wedel et al. [16], and Shore et al. [18] have criticized the manner in which policy studies have often presented policy as orderly, leaving out the messiness, the unpredictability and the disorderly elements of policy processes. Our material speaks to the disordered and rather confusing articulation between law, policy and practice, a messy scenario with implications for access. In the case of abortion policy and practice, key dynamics behind the messiness has to do with the fundamental dilemma of recognizing that induced abortions are carried out whatever the law says, hence allowing for the existence of arenas where abortions can take place invisibly and relatively safely, and without having to take up a morally impossible position of publicly fighting for rights to legal abortion.

With a legal framework that allows abortion on broad grounds, and a political environment dominated by anti-abortion discourse, Zambia is a good example of the messiness of policy processes. Informants explained how the law text itself provides important barriers to access through the request for the three signatures. But these barriers can sometimes be partly mitigated for example by the presence of Chinese clinics operating outside the legal framework or the recent increase in access to Misoprostol and combination packs for medical abortion over the counter for abortion-seeking women and girls in urban centres.

Within the highly restrictive legal abortion context in Tanzania, we found a complex discursive abortion landscape and a lack of systematic or large scale prosecution of abortion seekers and providers. This seemed to leave substantial room for health worker discretion, and allowed abortion services to be provided clandestinely by both trained and untrained individuals. Public post-abortion care services were available to prevent complications. The burgeoning illegal market for Misoprostol sold off label moreover opened access to medical abortion for urban women.

In the far more permissive legal context in Ethiopia, linked to the power vested in the claims of the woman, clinical guidelines have been developed to guide safe abortion procedures, health workers increasingly receive training, and services are gradually rolled out to the population. However, rather than encountering a context of ready access to safe abortion services for those who fulfill the criteria in the law, our material indicates that a number of factors continue to seriously limit access. The government’s fear of informing the public about the law so as not to appear as a state promoting induced abortion which could cause uproar at grassroots level, has caused information to be held back, limiting both knowledge about the law and the full roll-out of services. Even when public services are available, women may refrain from using them for the fear of disclosure in the community, while health workers act as gate keepers and may dismiss women because of religious conscience. The increasing availability of safe abortion services thus to some extent remains silenced, the law is not widely known, and high numbers of young women continue to resort to unsafe abortion procedures [47, 48]. Despite the slow progress, a relatively liberal law is long since in place, there is a careful but steady roll out of abortion services, and there is an acceptance for the increasing availability of Misoprostol and emergency contraception. These factors all speak to the continuous but silent attempts to expand the services in a context of massive anti-abortion sentiments.

What we encountered was a relationship between abortion-related policy- and access scenarios that emerged as paradoxical, where an inherent aspect of the ambiguity and messiness opened up for a substantial degree of political pragmatism. We found that even the most conservative and restrictive contexts to some extent, ultimately allowed avenues of access to abortion services to exist or operate. We encountered governments that quietly accepted that clandestine abortion services operated; governments that did not systematically prosecute illegal abortion seekers, abortion providers or vendors of illegal abortion drugs, and religious leaders who refrained from fights against the liberalization of the law. These paradoxical scenarios all suggest a pragmatic approach to implanting and enforcing abortion policies. The dynamics at work indicate that policy makers, religious leaders and other key actors in the field pragmatically manoeuvre between ways of relating to a highly stigmatized public health challenge that causes suffering and death among vast numbers of girls and women in their communities and their own desire to remain morally clean. The articulation between the strong public condemnation of abortion encountered at all levels in the three countries and the pragmatic stance towards loop holes in the system most powerfully surfaces through the rapidly increasing availability and accessibility of medical abortion drugs [49].

### Concluding remarks

In this comparative research project in Tanzania, Zambia and Ethiopia, we encountered a seemingly paradoxical relationship between national abortion laws, abortion policy and women’s actual access to abortion services. We do not question the close relationship between restrictive abortion laws, illegal and unsafe abortion and high maternal mortality scenarios documented globally. We do however, based on the material in this study, wish to contribute modestly to the literature that cautions against a too narrow focus on the content of legal and policy documents in assessment of outcomes, in this case the content of abortion laws versus access to abortion services. Through an analysis of contexually embedded actors differently situated vis-à-vis abortion policy and process, we can gain a more credible picture of actual access to safe abortion services in a given context. This implies being open to the messiness of policy processes. In our material the messiness has revealed a pragmatism characterizing even the most restrictive abortion contexts, a pragmatism likely to be linked to a realization that no law can prevent abortions from taking place. The pragmatism is particularly visible through the burgeoning Misoprostol market. This market, although hampered by general challenges linked to the marketing of drugs in low income contexts, entails considerable hope and anticipation through the drug’s ability to bypass both restrictive abortion laws and health workers as gate-keepers of access to safer abortion services.

### Study strengths and limitations

Studying law and policy processes calls for particular researcher reflexivity not the least because of the immense complexity of the topic. The research team recognizes the substantial limits to awareness given the particular constraints imposed by the controversial and sensitive nature of the study topic. This said, we believe that a comparative case study approach, including different country contexts, was fruitful in disentangling findings with increased transfer value. The quality of the study was, we believe, strengthened by the inclusion of informants differently postioned vis-à-vis the study topic. Finally, we think that a team of national and foreign researchers, schooled in the critical social sciences, and with substantial ethnographic research experience from the same African contexts, enhanced the quality of the research material.

## Data Availability

The interview data generated and analyzed during the current study is not publicly available to ensure the study participants’ anonymity. The data will be available from the corresponding author on reasonable request.
